# Decreased physical performance despite objective and subjective maximal exhaustion in post-COVID-19 individuals with fatigue

**DOI:** 10.1186/s40001-023-01274-5

**Published:** 2023-08-26

**Authors:** Shirin Vollrath, Lynn Matits, Jana Schellenberg, Johannes Kirsten, Jürgen M. Steinacker, Daniel A. Bizjak

**Affiliations:** 1 Division of Sports and Rehabilitation Medicine, Department of Medicine, Ulm University Hospital, Ulm, Germany; 2 Division of Clinical & Biological Psychology, Institute of Psychology and Education, Ulm University, Ulm, Germany

**Keywords:** Post-COVID-19, Fatigue, Physical exhaustion, Long COVID, COVID CPET, Physical exercise

## Abstract

**Introduction:**

Fatigue is a common symptom in post-COVID-19 patients. Individuals with fatigue often perform less well compared to healthy peers or without fatigue. It is not yet clear to what extent fatigue is related to the inability to reach maximum exhaustion during physical exercise.

**Methods:**

A symptom-based questionnaire based on the Carruthers guidelines (2003) was used for reporting the presence of fatigue and further symptoms related to COVID-19 from 85 participants (60.0% male, 33.5 ± 11.9 years). Cardiopulmonary exercise testing (CPET) and lactate measurement at the end of the test were conducted. Objective and subjective exhaustion criteria according to Wasserman of physically active individuals with fatigue (FS) were compared to those without fatigue (NFS).

**Results:**

Differences between FS and NFS were found in Peak V̇O_2_/BM (*p* < 0.001) and Max Power/BM (*p* < 0.001). FS were more likely to suffer from further persistent symptoms (*p* < 0.05). The exhaustion criterion Max. lactate was reached significantly more often by NFS individuals.

**Conclusion:**

Although the aerobic performance (Max Power/BM) and the metabolic rate (Peak V̇O_2_/BM and Max. lactate) of FS were lower compared to NFS, they were equally able to reach objective exhaustion criteria. The decreased number of FS who reached the lactate criteria and the decreased V̇O_2_ peak indicates a change in metabolism. Other persistent post-COVID-19 symptoms besides fatigue may also impair performance, trainability and the ability to reach objective exhaustion.

*Trial registration* Trial registration: DRKS00023717; date of registration: 15.06.2021 (retrospectively registered).

**Supplementary Information:**

The online version contains supplementary material available at 10.1186/s40001-023-01274-5.

## Introduction

The corona virus disease (COVID-19) has presented us with challenges far beyond those of an acute illness, similar to other pandemics like the “Spanish Flue”, the MERS- or Ebola epidemic [1, 2]. In some individuals, health consequences persist for a long time after COVID-19 [3–6]. The prevalence of individuals who suffer from persistent symptoms after acute infection ranges from 6.5% to almost 28.5% in the German working-age population [5]. Comorbidities increase the risk to suffer from persistent symptoms [7, 8]. Regardless of the fitness level, one of the most often reported symptoms is fatigue, which is associated with a drop in performance capacity [4, 5, 7, 9]. Individuals who reported having fatigue were also more likely to report chronic fatigue and/or rapid physical exhaustion [5]. Therefore, the question arises whether subjective fatigue can also be measured objectively and whether there is an objective correlation of fatigue and the ability to exhaust maximally.

Generally, cardiopulmonary exercise testing (CPET) is used to assess the exercise capacity and to examine the functions and interactions of the cardiac, muscular and respiratory components [10]. In addition, CPET offers the possibility to assess objective exhaustion parameters [11]. To our knowledge, there is no study investigating the objective exhaustion capacity in individuals with fatigue compared to individuals without fatigue based on a broad range of exhaustion variables. Therefore, this study addressed the question of whether the subjectively decreased performance of individuals with fatigue can be related to the inability to reach objective exhaustion criteria. The present study examined (1) possible differences in exhaustion variables according to Wasserman [11] between physically active post-COVID-19 individuals with and without fatigue symptoms; (2) whether individuals with fatigue symptoms (FS) were able to reach total physical exhaustion compared to peers without fatigue symptoms (NFS); and (3) whether the number of reached exhaustion criteria were different between FS and NFS individuals.

## Material and methods

### Study population and investigation period

The study was conducted at the Division for Sports and Rehabilitation Medicine, Center of Internal Medicine of the University Hospital in Ulm, Germany, between June 2020 and March 2022. The participants were recruited consecutively by the outpatient clinic of this division. The inclusion criteria were (1) age ≥ 18 years during the investigation period; (2) participation in sport at least 3 times per week (20 metabolic equivalents (METs)/week); (3) confirmed SARS-CoV-2 infection but at least > 2 weeks after a positive PCR test. In total 155 individuals were included and 85 individuals (60.0% male) had performed a CPET and had a lactate sample at the end of the CPET. Data collection took place on the same day as the study inclusion (4.4 ± 4.6 months after infection). Athletes were advised to follow current return to sport guidelines at this time [12, 13].

### Examination of symptoms

The information of the presence of fatigue was collected during the screening of their medical history based on the consensus criteria by Carruthers et al. [14]. Fatigue was considered as present if at least one of the fatigue symptoms (unexplainable performance decrease, persistent mental or physical fatigue, prolonged regeneration, worsening of symptoms after physical exertion) was reported to be new after COVID-19. When fatigue was present before COVID-19 the participant was excluded from the analysis. Based on their answers they were divided in two groups: fatigue symptoms (FS) and not fatigue symptoms (NFS). All participants, except one who was hospitalized due to the acute COVID-19, had mild–moderate disease courses. Table 1 shows the anthropometric data, symptoms during acute period, sport type and training volume before disease, further persistent symptoms in addition to the symptom fatigue of the FS study population as well as lung restrictions at the examination. Sport type were classified in four different categories (1) endurance (e.g., running, biking, rowing); (2) resistance (e.g., general resistance training, fitness, Pilates); (3) team/combat sport (e.g., fencing, soccer, handball); (4) technique (e.g., gymnastics, sport aerobic).

**Table 1:** Anthropometric data, sport type, training volume before disease and persistent symptoms at investigation (*N* = 85) *p < 0.05, **p < 0.01, ***p = 0.001, **** *p* < 0.001

Anthropometric data	Fatigue symptoms (FS)	No fatigue symptoms (NFS)	Differences between both groups
	Mean ± standard deviation	Mean ± standard deviation	*p*-value (Cramer-V)
Age (years)	37.1 ± 12.5	30.0 ± 10.0	0.006**
Body mass (BM) (kg)	77.1 ± 16.9	75.5 ± 13.5	0.736
Height (cm)	174.9 ± 9.3	179.0 ± 8.5	0.037*
Body mass index (BMI) (kg/m^2^)	25.05 ± 4.0	23.6 ± 3.0	0.070
Sex	F: 21, M: 21	F: 13, M: 30	0.064
Time since infection (months)	6.3 ± 5.4	2.5 ± 2.6	< 0.001****
Type of sports^a^	Frequency	Frequency	
Endurance	29 (59.2%)	21 (42.0%)	
Resistance	7 (14.3%)	11 (22.0%)	
Team/combat	10 (20.4%)	16 (32.0%)	
Technique	3 (6.1%)	0	
Missing	0	2 (4.0%)	
Weekly training volume before disease	Number	Number	
3–5 h	26 (61.9%)	14 (32.6%)	
5–10 h	11 (26.2%)	16 (37.2%)	
10–15 h	1 (2.4%)	7 (16.3%)	
> 15 h	3 (7.1%)	6 (14.0%)	
Missing	1 (2.4%)	0	
Symptoms during acute phase^a^	Frequency	Frequency	
Fever (> 38 °C)	15	18	0.543 (0.037)
Cough	13	19	0.181 (0.161)
Ageusia/anosmia	15	19	0.398 (0.102)
Rhinitis	16	21	0.281 (0.130)
Throat pain	20	16	0.276 (0.131)
Dyspnea under load	19	10	0.022* (0.277)
Dyspnea at rest	12	4	0.019* (0.283)
Diarrhea	7	7	0.952 (0.007)
Headache	17	22	0.281 (0.130)
Missing	8	8	
Further persistent symptoms^a^	Frequency	Frequency	
Sleeping disorders	20	10	0.019* (0.255)
Neurocognitive disorders	28	9	< 0.001**** (0.461)
Respiratory disorders	21	5	< 0.001**** (0.416)
Autonomic disorders	23	9	0.001*** (0.349)
Muscle pain	14	4	0.007** (0.294)
Psychological-related items	1	0	0.314 (0.110)
Immunological disorders	8	1	0.012* (0.272)
No symptoms	0	22	
Lung involvement at examination			
Minimal abnormalities in body plethysmography	4 (9.5%)	2 (4.7%)	
Minimal abnormalities in spirometry	1 (2.3%)	1 (2.3%)	
Minimal abnormalities in diffusion capacity	7 (16.7%)	1 (2.3%)	
Abnormalities in body plethysmography	3 (7.1%)	1 (2.3%)	
Abnormalities in spirometry	4 (9.5%)	1 (2.3%)	
Abnormalities in diffusion capacity	2 (4.7%)	0	

^a^Multiple choice possible

### Examination of CPET and lactate determination

An individualized ramp protocol was used during CPET on the bicycle ergometer. Detailed information of this specific study execution is described in another research article [9]. The following variables which were used to determine objective exhaustion according to Wasserman [11] were determined: Peak V̇O_2_/BM (volume oxygen/body mass) (ml/min/kg BM), Max Power/BM (maximal power/BM) (Watt/kg BM), Peak HR (heart rate) (beats/min), peak breathing frequency (BF) (1/min), Peak V̇E/V̇O_2_ (peak ventilation/V̇O_2_) (l/min/l/min), Peak respiratory exchange ratio (RER) (V̇CO_2_/V̇O_2_) (l/min/l/min). Furthermore, the examiner visually assessed whether there was a plateau of the HF and Peak V̇O_2_/BM. The Perceived Subjective Muscular Exhaustion and Perceived Subjective General Exhaustion were determined on the Rate of Perceived Exertion scale (RPE scale, 0–10, no exhaustion – total exhaustion) after the ramp test. Capillary blood was taken from the ear lobe as soon as individuals or the examiner stopped the ramp test and the maximum lactate (Max. lactate) value was determined [15]. Missing values occurred due to measurement errors.

### Criteria for total exhaustion

The following criteria were applied to check if the individuals reached objective total exhaustion on the bicycle ergometer [11]:

Plateau of HR almost at the end of the test, reaching maximum HR (Maximum HR = rel. HR_max_ − 10 beats), plateau of V̇O_2_ almost at the end of the test, RER ≥ 1.15, BF > 50 at the end of the test, O_2_-ventilatory equivalent (V̇E/V̇O_2_) > 30–35, lactate criterion > 8 − 10 mmol/l, Perceived Subjective General Exhaustion (> 17 at BORG-Scale).

Not all criteria have to be reached by the individuals to be considered exhausted. However, individual exhaustion criteria that have not been reached can provide information about where patients have limitations [10]. Due to the physically active cohort, we assumed that the participants are used to high levels of exhaustion. Therefore, we applied the following criteria: V̇E/V̇O_2_ > 35, lactate > 10 mmol/l. The criterion for the Perceived Subjective General Exhaustion was adapted from the BORG-scale (6–20, really really easy–really really hard) to the RPE-Scale used here. Values ≥ 9 in the RPE scale were considered as total exhaustion. Rel. HR_max_ was calculated with the following formula: rel. HR_max_ = 208−(0.7∙Age), (rel.HR_max_ = relative maximal heart rate) [11].

### Statistical analysis

GraphPad Prism, version 9.4.1 (Dotmatics, Boston, USA) and IBM SPSS Statistics, version 28.0.0.0 (IBM Deutschland GmbH, Ehningen, Germany) were used for the statistical analysis. To calculate differences between training volume before disease, symptoms during acute phase as well as further persistent symptoms, Pearson Chi-square test and Cramer V were used. Adjusted significance level was *p* < 0.003 when considering multiple testing (Bonferroni correction). For determining the difference between FS and NFS in interval-scaled exhaustion variables, Welch’s *t*-tests or the Mann–Whitney *U* tests were used. To control for possible covariates (age, sex, body mass, height, BMI, time since infection and training volume), robust linear regression models were conducted separately for each covariate. To calculate whether there is a difference in medians between the numbers of reached exhaustion criteria by each individual, Mann–Whitney *U* tests were used. To determine whether there were differences between FS and NFS in the number of individuals who reached the corresponding exhaustion criterion, Chi-square tests and Cramer V were calculated. Adjusted significance level when considering multiple testing was *p* < 0.006 (Bonferroni correction). The general significance level for all tests was set at (*p* < 0.05).

## Results

### Differences between FS and NFS regarding symptoms

Of all 85 individuals, 42 FS participants (21 females) and 43 NFS participants (13 females) were identified. FS participants (37.1 ± 12.5 years) were ~ 7 years older than NFS participants (30.0 ± 10.0 years). The age difference is significant, but it only influences the group difference in Peak HR. The time since infection differs between FS (6.3 ± 5.4 months) and NFS (2.5 ± 2.6 months). In both groups, endurance sports were named most frequently. There was a significant correlation between FS/NFS group affiliation and the pre-disease training volume, indicating that NFS individuals had a higher pre-disease training volume (*p* < 0.001, *τ* = − 0.303). FS participants were more likely to have dyspnea under load (*p* = 0.022, *V* = 0.277) and rest (*p* = 0.019, *V* = 0.283) during acute phase than NFS participants. When multiple testing was conducted, these differences did not remain. FS participants reported significantly more persistent symptoms (sleeping disorders (*p* = 0.019, *V* = 0.255), neurocognitive disorders (*p* < 0.001, *V* = 0.461), respiratory disorders (*p* < 0.001, *V* = 0.416), autonomic disorders (*p* = 0.001, *V* = 0.349), muscle pain (*p* = 0.007, *V* = 0.294), and immunological disorders (*p* = 0.012, *V* = 0.272). After correction for multiple testing, neurocognitive disorders, respiratory disorders and autonomic disorders were still significant. Psychological-related items did not differ significantly. Overall, 22 NFS participants reported that they had no persistent symptoms.

### Differences between FS and NFS regarding exhaustion variables

**Fig. 1:**
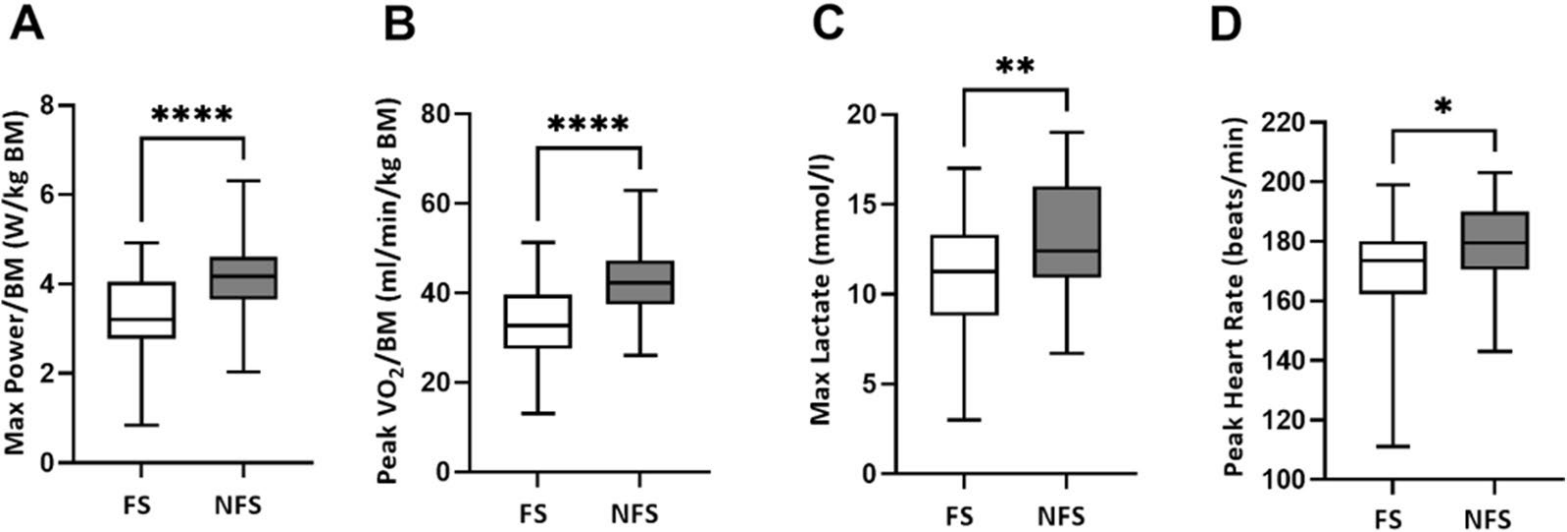
Distribution of performance and exhaustion values between *FS* (Fatigue symptoms) and *NFS* (No fatigue symptoms) **A** Max Power/BM (*p* < 0.001), **B** Peak V̇O_2_/BM (*p* < 0.001), **C** Max lactate (*p* = 0.008) and **D** Peak HR (*p* = 0.022). *BM* body mass, *V̇O*_*2*_ volume oxygen, *HR* heart rate. Differences between both groups are shown without considering covariates. Differences marked with *. *p < 0.05, **p < 0.01, **** *p* < 0.0001

Differences between FS and NFS were observed for the performance variables Max Power/BM (FS: 3.29 ± 0.91, NFS: 4.20 ± 0.77, *p* < 0.001) and Peak V̇O_2_/BM (FS: 33.07 ± 7.75, NFS: 41.93 ± 7.46, *p* < 0.001) (Fig. 1). When compared to the NFS group, Max lactate and Peak HF levels were lower in FS (FS: 11.07 ± 3.31, NFS 13.01 ± 3.23, *p* = 0.008 and FS: 170.30 ± 17.81, NFS: 179.20 ± 113.36, *p* = 0.022, respectively). However, the group differences in Max lactate did not remain significant when controlling for the covariate time since infection (*p* = 0.121). Group differences in Peak HF did not remain significant when controlling for age (*p* = 0.401) and time since infection (*p* = 0.115). The variables Peak RER, Peak V̇E/V̇O_2_, Peak BF, Perceived Subjective General Exhaustion, and Perceived Subjective Muscular Exhaustion did not differ significantly between the two groups (Additional information about the influence of the confounder on the correlation between both groups for the variables Peak V̇O_2_/BM, Max Power/BM, Max. lactate, Peak HR are shown in Additional file 1: Table S1).

### Determination of total exhaustion

Table 2 shows the number of individuals reaching exhaustion criteria, according to their respective groups. NFS individuals met exhaustion criteria more frequently, with the exception of Peak V̇E/V̇O_2_ and the Perceived Subjective General Exhaustion. A significant difference was found for Max lactate. NFS participants reached the 10 mmol/l criterion more often. After correction for multiple testing, the difference in Max lactate between both groups was no longer significant.

**Table 2:** Number, percentage and differences of exhaustion criteria reached by individuals in the FS (fatigue symptoms) (*N* = 42) and NFS (no fatigue symptoms) (*N* = 43) groups

Exhaustion criterion	Absolute number of individuals who reached the exhaustion criterion (relative number in %)		Differences between both groups
	Fatigue symptoms	No fatigue symptoms	*p*-value (Cramer-V)
Plateau of HR	5 (13.5%)	6 (**16.2%**)	0.744 (0.038)
Reaching maximum HR	16 (43.2%)	20 (**55.6%**)	0.346 (0.111)
Plateau of V̇O_2_	10 (26.3%)	12 (**30.8%**)	0.665 (0.049)
Peak RER ≥ 1.15	29 (74.4%)	35 (**89.7%**)	0.077 (0.200)
Peak VE/ V̇O_2_ ≥ 35	28 (**71.8%**)	27 (69.2%)	0.804 (0.028)
Peak BF ≥ 50	4 (10.3%)	7 (**17.9%**)	0.329 (0.111)
Max. lactate > 10 mmol/l	26 (61.9%)	37 (**86.0%**)	0.011* (0.276)
Perceived subjective general exhaustion ≥ 9	33 (**82.9%**)	30 (76.9%)	0.412 (0.092)

The percentage of the group that reached the criteria more frequently and significant differences between groups are shown in bold

*p < 0.05

*HR* heart rate, *V̇O*_*2*_ volume oxygen, *RER* respiratory exchange ratio, *BF* breathing frequency, *V̇E* ventilation

### Number of exhaustion criteria reached per participant

The analysis included 31 FS participants and 30 NFS participants. The missing data were due to individual missing values. The mean number of exhaustion criteria was three for FS and four for NFS. However, the number of exhaustion criteria were not significantly different (*T*: 2.157, *p* = 0.142).

## Discussion

In our study, we found that FS had lower physical performance (decreased Max Power/BM) and a reduced metabolic rate (decreased Peak V̇O_2_/BM and maximal lactate levels) than NFS. However, they were able to reach exhaustion criteria to the same extent as individuals of NFS, with the exception of Max. lactate. Additionally, individuals with fatigue symptoms reported significantly more often dyspnea during acute phase and further persistent symptoms.

### Differences in FS and NFS between symptoms

In total, 61.8% of the investigated females and only 41.1% of males reported having fatigue symptoms. This result is in line with findings by Peters et al. [5] who showed that women of working age documented substantial or extreme fatigue after COVID-19 more frequently than men. Additionally, FS individuals were older than NFS individuals. This is in line with previous findings of studies which identified age as a risk factor for suffering from persistent symptoms [16, 17]. Crook et al. [18] attributed fatigue to increased neuroinflammation and decreased function of neuromuscular synapses, but also clumping within the body and injury to the endothelium and chronic inflammation can be triggers of fatigue [18].

### Differences between FS and NFS in exhaustion variables

Differences between FS and NFS were found in Peak V̇O_2_/BM and Max Power/BM. This result is partly in line with the findings in athletes after COVID-19 with a mild-to-moderate disease course by Anastasio et al. [19]. V̇O_2_ was decreased at the aerobic threshold compared to non-infected controls. In contrast to our study, they did not report differences in peak power. In a previous study (partly overlapping with this study population), we found that the existence of persistent symptoms in athletes was associated with a decreased maximal power and V̇O_2_ peak compared to those who were symptom-free [9]. All these study results indicate that the disease itself, the existence of persistent symptoms as well as the symptom fatigue reduce the physical performance of individuals.

In the present study, we observed decreased Peak HR and Max lactate in FS compared to NFS, when confounders were not considered. In contrast to our study, Romero-Ortuno et al. [20] found that reaching a target of 85% of maximal heart rate became more likely the longer the period between infection and examination. However, these individuals were older and were more frequently hospitalized than our study participants. Another study showed a longitudinal decrease in HR peak over a period of 3 months in athletes with persistent symptoms, including exercise intolerance and fatigue [21]. Nevertheless, comparison between study results should be considered with caution as a control for age and its effect on Peak HR should be done in each study.

### Total exhaustion and number of reached exhaustion criteria

Physical exhaustion can be described as a subjective feeling in CPET and evaluated with objective exhaustion criteria. In our study, 82.9% individuals of FS and 76.9% individuals of NFS reported total exhaustion at the end of the test. We found that FS, compared to NFS, were also able to reach the objective exhaustion criteria by Wasserman et al. [11], with the exception of Max lactate. The decreased maximal lactate accumulation in FS may indicate a lower metabolic capacity. Lactate is an energetic muscular fuel which can be oxidized correspondently better in oxidative muscles fibers than glycolytic fibers [22]. The circulating lactate is a supporter of the NADH electron flux into mitochondria [23]. Furthermore, it is maybe an indirect indicator of activity of the mitochondrial pyruvate dehydrogenase complex (Complex I) [23]. Therefore, a decreased circulating lactate may indicate reduced delivery of mitochondrial energy flux which corresponds to a lower V̇O_2._

In blood serum, an altered fatty acid metabolisms and a lower lactate accumulation was observed at rest in Long-COVID patients, compared to healthy participants [24]. They concluded that the disturbed fatty acid metabolism is one reason for exercise intolerance among Long-COVID patients. Further studies showed impaired mitochondria in COVID-19 patients, which could be a further explanation of a decreased performance [25]. All in all, these results may indicate that the lactate metabolism may be altered, but further studies should investigate the role of exercising, fatigue and the duration of the disease.

The RER did not differ significantly in our study and the results of other studies on RER vary. Moulson et al. [21] showed that athletes with cardiopulmonary symptoms and healthy athlete controls have no differences in RER. However, in another study the number of patients with post-COVID-19 syndrome reaching the anaerobic threshold was significantly lower than patients who were already symptom-free [26]. These contrasting findings suggest that outcomes may depend on the fitness of the study population and the severity of symptoms, among other factors.

We observed a decreased BF in FS and NFS. Parkes et al. [27] observed a decreased mean value of BF among Long-COVID patients, and Loew et al. [28] found different types of dysfunctional breathing in patients with mostly mild acute phases. These results may partly explain the decreased BF in our study population, but it might also have other possible explanations than fatigue like detraining or disturbed oxygen supply, because NFS also had a decreased breathing frequency.

## Strengths and limitations

To our knowledge, there is no study which evaluated the objective exhaustion criteria in a physically active cohort with fatigue symptoms. In addition, fatigue is a multidimensional symptom with often accompanying symptoms. To be able to clarify this disease pattern, further studies should be conducted. Age not only influences the risk of fatigue, it can also affect performance, ventilation and may influence the ability to reach maximal exhaustion. The influence of age was considered for the exhaustion variables but general influences should be considered in more detail. The difference in the time period between infection and examination between the groups may have skewed the results and could be due to the fact that people suffering from fatigue enrolled later in the study because of fatigue, while people without persistent symptoms wanted to resume their training as soon as possible and therefore came earlier. This could lead to muscle loss and general deconditioning. Therefore, it can be assumed that the detected performance loss is a result of persistent symptoms, general deconditioning and further factors, like an impaired lactate metabolism. Upcoming studies should evaluate these issues, to determine their proportions at the performance loss. To analyze the extent of deconditioning studies which compare the pre- and post-infection status are needed. Moreover, the study population was too small to conduct analysis with subgroups and there were no pre-disease data.

Moreover, different types of sport, the duration of training session and the intensity affect the production / removal of lactate [29]. Therefore, the difference in reaching the lactate criterion by the individuals cannot fully explained. In addition, the inclusion criterion fatigue in FS may has to many severity levels, within this group and deconditioning in the acute phase can lead to rapid fatigue, and rapid fatigue can lead to further deconditioning. Therefore, the origin of fatigue is not always determinable.

## Conclusion

Aerobic performance (Max Power/BM and Peak V̇O_2_/BM) of FS was lower compared to NFS after COVID-19. However, they were equally able to reach objective exhaustion criteria. When individuals suffer from fatigue symptoms, they often have other persistent symptoms like respiratory disorders or neurocognitive impairment, which can also negatively affect performance and the reduced training volume due to persistent symptoms probably leads to further deconditioning. However, we showed also initial indicators of an altered lactate metabolism in individuals with persistent fatigue that might contribute to decreased performance and increased severe fatigue and/or diagnosed post-exertional-malaise. mitochondrial (e.g., OxPhos and morphology) and lactate (e.g., kinetics during CPET, lactate metabolites) diagnostics may add valuable insights into the fatigue-related mechanism in athletes after a COVID-19 infection. In summary, the infection may cause an initial decline in performance. However, the evolution of performance depends on the presence and severity of persistent symptoms and the mechanisms that cause the symptoms.

### Supplementary Information

**Additional file 1: Table S1.** Influence of confounders on the correlation between both groups for the variables Peak V̇O2/BM (Volume Oxygen/Body Mass), Max Power/BM (Maximal Power/BM), Max. lactate (maximum lactate), Peak HR (Peak Heart Rate).

## Data Availability

The datasets generated and/or analyzed during the current study are not publicly available because it is critical patient data but are available from the corresponding author on reasonable request.

## Abbreviations

BF: Breathing frequency
CPET: Cardiopulmonary exercise testing
COVID-19: Corona virus disease-19
FS: Physically active individuals with fatigue symptoms
HR: Heart rate
Max. lactate: Maximum lactate
Max Power/BM: Maximum power/body mass
ME/CFS: Myalgic encephalomyelitis/chronic fatigue syndrome
MET: Metabolic equivalents
NFS: Physically active individuals with no fatigue symptoms
RER: Respiratory exchange ratio
rel.HR_max_: Relative maximal heart rate
RPE scale: Rate of Perceived Exertion Scale
V̇E/V̇O_2_: O_2_-ventilatory equivalent
V̇O_2_: Volume oxygen
V̇O_2_/BM: Volume oxygen/body mass
