# Supplementary material for: Decreased physical performance despite objective and subjective maximal exhaustion in post-COVID-19 individuals with fatigue
Source: Eur J Med Res. 2023 Aug 26;28:298. doi: 10.1186/s40001-023-01274-5 (PMC10464445; doi:10.1186/s40001-023-01274-5)
Supplement: Supplementary file 1 — Additional file 1: Table S1. Influence of confounders on the correlation between both groups for the variables Peak V̇O2/BM (Volume Oxygen/Body Mass), Max Power/BM (Maximal Power/BM), Max. lactate (maximum lactate), Peak HR (Peak Heart Rate). [file 40001_2023_1274_MOESM1_ESM.docx]

**Additional File**

Table 1. **Influence of confounders on the correlation between both groups** for the variables Peak V̇O_2_/BM (Volume Oxygen / Body Mass), Max Power/BM (Maximal Power / BM), Max. lactate (maximum lactate), Peak HR ( Peak Heart Rate).

| **Confounder** | **Peak V̇O_2_/BM** (ml/min/kg BM) | | **Max Power/BM** (Watt/kg BM) | | **Max. lactate** (mmol/l) | | **Peak HR**  (beats/min) | |
| --- | --- | --- | --- | --- | --- | --- | --- | --- |
|  | **T** | **p-value** | **T** | **p-value** | **T** | **p-value** | **T** | **p-value** |
| **Training volume (hours)** | 3.75 | **<0.001** | 4.07 | **<0.001** | 2.74 | **0.008** | 2.30 | **0.025** |
| **Age (years)** | 4.29 | **<0.001** | 4.13 | **<0.001** | 2.06 | **0.042** | 0.85 | 0.401 |
| **Body mass (kg)** | 4.92 | **<0.001** | 4.88 | **<0.001** | 2.80 | **0.006** | 2.33 | **0.023** |
| **Height (cm)** | 3.90 | **<0.001** | 4.37 | **<0.001** | 2.37 | **0.020** | 2.15 | **0.035** |
| **BMI (kg / m^2^)** | 4.71 | **<0.001** | 4.47 | **<0.001** | 2.99 | **0.004** | 2.17 | **0.033** |
| **Time since infection (months)** | 3.98 | **<0.001** | 4.08 | **<0.001** | 1.57 | 0.121 | 1.60 | 0.115 |
| **Sex** | 4.31 | **<0.001** | 4.48 | **<0.001** | 2.22 | **0.029** | 2.33 | **0.023** |

T (standardized test statistic), p-value <0.05 (significance level), BM = body mass
